# Supplementary material for: From sequence to enzyme mechanism using multi-label machine learning
Source: BMC Bioinformatics. 2014 May 19;15:150. doi: 10.1186/1471-2105-15-150 (PMC4229970; doi:10.1186/1471-2105-15-150)
Supplement: Additional file 2 — Java code of ml2db. Additional file ml2db_code.tar.gz contains the Java source code to run the multi-label machine learning experiments and save the results to database. The code’s Javadoc is included. [file 1471-2105-15-150-S2.zip › additional file 2/ml2db/ml2db/doc/index-files/index-2.html]

B-Index


JavaScript is disabled on your browser.


- Overview
- Package
- Class
- Use
- Tree
- Deprecated
- Index
- Help

- Prev Letter
- Next Letter

- Frames
- No Frames

- All Classes

A B C D E F G H I J K L M N O P Q R S T U V W X 


## B

BASE\_ATTRIBUTE\_SHAPE - Static variable in class main.ml2db.graphviz.GraphVizArff


BASE\_CLASS\_SHAPE - Static variable in class main.ml2db.graphviz.GraphVizArff


BASE\_INSTANCE\_SHAPE - Static variable in class main.ml2db.graphviz.GraphVizArff


BASE\_PATH - Static variable in class test.ml2db.dataharness.TestProjectParameters


BINARY\_ATTRIBUTE\_ABSENT\_VALUE - Static variable in class main.ml2db.weka.AttributeFactory


BINARY\_ATTRIBUTE\_PRESENT\_VALUE - Static variable in class main.ml2db.weka.AttributeFactory


bipartitionsToString(TreeMap<String, boolean[]>) - Method in class main.ml2db.mulan.measuremetrics.AnnotationsReader


BRkNNALTest - Class in test.ml2db.mulan.classifier.lazy
:   Class

BRkNNALTest() - Constructor for class test.ml2db.mulan.classifier.lazy.BRkNNALTest


BRkNNFewInstances - Class in main.ml2db.mulan.classifier.lazy
:   An implementation of Mulan BRkNN for Active Learning that allows predictions
    confidence to raise above 50% even when the instances in the training set are
    fewer than (labels - 1).

BRkNNFewInstances(int) - Constructor for class main.ml2db.mulan.classifier.lazy.BRkNNFewInstances


BRkNNVisible - Class in main.ml2db.mulan.classifier.lazy
:   A version of BRkNN Mulan 1.4 that exposes more methods as public, and has
    more logging and debugging capabilities.

BRkNNVisible() - Constructor for class main.ml2db.mulan.classifier.lazy.BRkNNVisible
:   Default constructor

BRkNNVisible(int) - Constructor for class main.ml2db.mulan.classifier.lazy.BRkNNVisible
:   A constructor that sets the number of neighbors

BRkNNVisible(int, BRkNNVisible.ExtensionType) - Constructor for class main.ml2db.mulan.classifier.lazy.BRkNNVisible
:   Constructor giving the option to select an extension of the base version

BRkNNVisible.ExtensionType - Enum in main.ml2db.mulan.classifier.lazy
:   The two types of extensions

buildAndSerialize() - Method in class main.ml2db.mulan.learn.MulanSerializer


ByFrequencyPicker - Class in main.ml2db.al.utility.pickers.byfrequency
:   Picks each of the features (class, attribute or attribute set) by descending
    order of frequency, and then, uniformly randomly, one of the instances of
    that feature.

ByFrequencyPicker() - Constructor for class main.ml2db.al.utility.pickers.byfrequency.ByFrequencyPicker

A B C D E F G H I J K L M N O P Q R S T U V W X

- Overview
- Package
- Class
- Use
- Tree
- Deprecated
- Index
- Help

- Prev Letter
- Next Letter

- Frames
- No Frames

- All Classes
